# Treatment of Periorbital Edema in a Patient With Systemic Lupus Erythematosus During Pregnancy: A Case Report Written With the Assistance of ChatGPT

**DOI:** 10.7759/cureus.36302

**Published:** 2023-03-17

**Authors:** Jacqueline Jansz, Michael J Manansala, Nadera J Sweiss

**Affiliations:** 1 Internal Medicine, University of Illinois at Chicago, Chicago, USA; 2 Rheumatology, Rush University Medical Center, Chicago, USA; 3 Rheumatology, University of Illinois at Chicago, Chicago, USA

**Keywords:** chatgpt, pregnancy, sun exposure, periorbital edema, sle, systemic lupus erythematosus

## Abstract

Systemic lupus erythematosus (SLE) is an autoimmune disease that has a wide range of manifestations and can affect nearly every organ system. Skin manifestations are a common finding in SLE. They are often photosensitive and can be exacerbated by exposure to ultraviolet light. Here, we discuss the case of a 34-year-old African American woman who presented with periorbital edema while 12 weeks pregnant. This case highlights the importance of avoiding sun exposure in patients with SLE and the challenge of treating SLE during pregnancy.

## Introduction

Systemic lupus erythematosus (SLE) is a chronic autoimmune disease that can affect various organs and systems in the body. It occurs when the immune system attacks healthy tissues, causing inflammation and damage. While the exact cause of SLE is unknown, genetics, hormones, and environmental factors are believed to play a role [1].

Dermatologic findings are common in SLE, with 85% of patients experiencing skin involvement at some point in the course of the disease [2]. Photosensitivity, or increased sensitivity to sunlight and other sources of ultraviolet (UV) radiation, is a common feature of SLE and can exacerbate skin symptoms [3].

The most common dermatologic manifestations in SLE are a malar rash (a butterfly-shaped rash over the cheeks and nose), discoid rash (scaly, red patches that can lead to scarring), and photosensitive rash (a rash that appears on sun-exposed skin) [4]. Other skin findings in SLE may include oral ulcers, alopecia, and Raynaud’s phenomenon (a condition where the fingers and toes turn white or blue in response to cold or stress) [4].

The management of dermatologic findings in SLE typically involves avoiding sun exposure, using a broad-spectrum sunscreen, and wearing protective clothing. Topical and systemic medications, such as corticosteroids and antimalarials, may also be used to treat skin symptoms [5]. Close monitoring and management of SLE by a healthcare professional are essential to prevent complications and improve quality of life.

## Case presentation

A 34-year-old African American woman with a past medical history significant for SLE, class V lupus nephritis, and hypothyroidism presented to the rheumatology clinic for evaluation of new periorbital swelling. At the time of the presentation, she was 12 weeks pregnant. She was originally diagnosed with SLE two years prior when she presented with fatigue and pancytopenia. Her serologies were notable for antinuclear antibody 1:1280 (<1:80), anti-dsDNA 1:160 (<1:10), +antinuclear ribonucleoprotein, +anti-Smith, +anti-RO, and +anti-La. Class V lupus nephritis was diagnosed on kidney biopsy and was notable for immune complex deposition consistent with membranous lupus nephritis. The periorbital edema was severe and limited her ability to see. She reported difficulty opening her eyes because of the edema, but denied any other visual changes. She denied any other areas of swelling, rash, or joint pain. She did not have any recent exposure to allergens or new facial products. She was maintained on a regimen of azathioprine 75 mg daily and hydroxychloroquine 400 mg daily.

Examination revealed bilateral periorbital and facial edema without redness, and periorbital cellulitis was excluded. Her labs at the time were notable for C3 63 (90-180 mg/dL) and leukopenia (white blood cell count of 2.0 K/µL). Thyroid antibodies were elevated to 416.4 (<9 IU/mL), but thyroid-stimulating hormone (TSH) was 2.84 (0.35-4 µIU/mL). The periorbital edema in the setting of leukopenia and hypocomplementemia were concerning for an SLE flare. Azathioprine was discontinued given her leukopenia, and she was started on prednisone 10 mg daily. She responded well to this regimen, and on follow-up two weeks later, had near resolution of the periorbital edema.

One week after the near resolution of her periorbital edema, she presented again with worsening facial swelling, as well as a rash on her face, neck, and chest. Her symptoms began after she returned from a vacation. During her vacation, she had prolonged sun exposure. Her physical examination was notable for diffuse facial and periorbital edema. There was a malar rash with underlying erythema and areas of dry crusting and yellow discharge. There was also an erythematous rash with scattered plaques on the sun-exposed areas of her neck and upper chest. The patient was admitted to the hospital for intravenous (IV) steroids and treatment of facial cellulitis.

**Figure 1:**
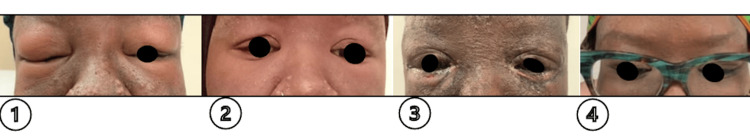
Periorbital edema in a pregnant patient with systemic lupus erythematosus. (1) Initial presentation: started on prednisone 10 mg. (2) Two weeks after starting prednisone 10 mg. (3) Presentation on admission to the hospital after prolonged sun exposure. (4) Presentation four weeks after hospital admission.

During her hospital admission, her labs were significant for an elevated erythrocyte sedimentation rate of 100 (<20mm/hour), C-reactive protein of 23 (<8.0 mg/L), and dsDNA (1:160), and a culture of the facial rash was positive for *Staphylococcus aureus*. She was started on IV methylprednisolone 40 mg for the SLE flare. This was transitioned to 30 mg of prednisone daily and then tapered down to 10 mg over the course of two weeks. She was treated with IV clindamycin and then transitioned to amoxicillin for a total of seven days of antibiotics for possible facial cellulitis. She was discharged after seven days in the hospital with marked improvement in facial swelling, rash, and cellulitis (Figure 1).

## Discussion

Periorbital edema, or swelling around the eyes, is an uncommon symptom in SLE and can be particularly challenging to treat in pregnant patients due to the limitations on medication use during pregnancy. In this case report, we demonstrate the efficacy of low-dose prednisone and the importance of skin-protective measures.

In this case, one of the main challenges is balancing the need for effective treatment with the safety of the developing fetus. Many medications used to treat periorbital edema, such as non-steroidal anti-inflammatory drugs, are associated with potential risks to the fetus and may be contraindicated or require close monitoring during pregnancy [6].

Our patient was initially treated with low-dose prednisone with excellent response. However, she then had prolonged sun exposure which resulted in a second flare-up of her skin requiring hospital admission. The second flare occurred while the patient was on prednisone 10 mg and required escalation to IV methylprednisolone as well as antibiotics to treat a possible skin infection.

Another challenge is the need to differentiate between periorbital edema caused by SLE and other causes of eye swelling that may be more common in pregnancy, such as preeclampsia. This requires close collaboration between a multidisciplinary team of healthcare professionals, including an obstetrician, a rheumatologist, and an ophthalmologist.

This patient’s periorbital edema with associated photosensitive rash was distinguished from other causes, including hypothyroidism, nephrotic syndrome, or preeclampsia. The periorbital edema in this case could not be explained by nephrotic syndrome alone. Additionally, she was normotensive which makes both nephrotic syndrome and preeclampsia less likely. Although she had elevated thyroid antibodies, which can be a cause of periorbital edema, her TSH was within normal limits, and she was receiving levothyroxine. Lastly, it is unlikely that any of these other conditions would present with a photosensitive rash.

Sun protection is crucial for individuals with SLE due to their heightened sensitivity to UV radiation from the sun [3]. Exposure to UV radiation can trigger or exacerbate SLE symptoms such as rashes, joint pain, and fatigue [7]. In addition, UV radiation can cause skin damage and increase the risk of skin cancer in individuals with SLE [8]. Therefore, it is recommended that individuals with SLE take extra precautions to protect themselves from the sun, including wearing protective clothing, applying sunscreen with a high SPF, avoiding direct sunlight during peak hours, and seeking shade whenever possible. By practicing sun safety, individuals with SLE can manage their symptoms and maintain overall skin health.

Additionally, managing SLE during pregnancy requires close monitoring of disease activity, as flares can lead to complications such as preterm labor and fetal growth restriction [9]. This may require adjustments to medication regimens or more frequent monitoring, which can be particularly challenging in patients with periorbital edema who may have difficulty seeing and performing daily activities.

Overall, the management of periorbital edema in pregnant patients with SLE requires a careful balancing of risks and benefits, close collaboration between multiple specialties, and individualized treatment plans based on the specific needs and circumstances of the patient.

**Figure 2:**
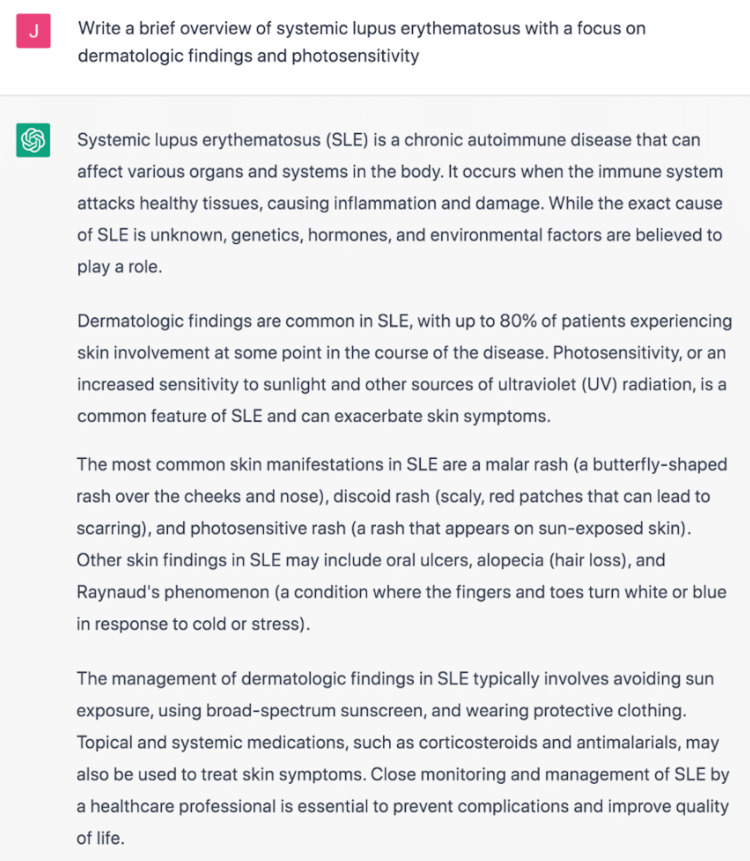
ChatGPT query to obtain the introduction.

**Figure 3:**
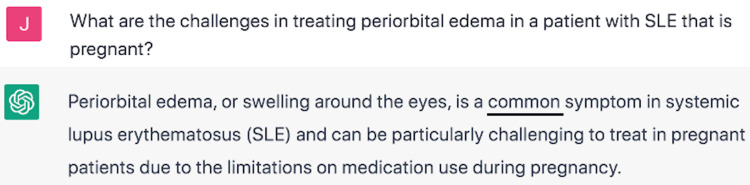
Incorrect statement by ChatGPT.

ChatGPT is an artificial intelligence product of OpenAi that simulates conversations with humans. It has been touted as being able to write poetry, essays, emails, and answer questions. However, can it be used as an aide in writing case reports? ChatGPT was able to summarize a topic well. However, it did not incorporate citations from relevant literature (Figure 2). ChatGPT also incorrectly called periorbital edema in SLE a “common” symptom when it is in fact uncommon (Figure 3). Additionally, ChatGPT is only knowledgeable about topics up to the year 2021, limiting its ability to comment on recent literature. ChatGPT is a useful tool, but experts are still required to proofread and cite relevant literature when writing using ChatGPT. In the future, hopefully, ChatGPT and other chatbots will have the ability to more accurately cite literature and provide resources.

## Conclusions

Lupus erythematosus is an autoimmune disease with a wide spectrum of clinical manifestations. Although many factors can trigger a flare, arguably one of the most important is UV radiation. This case demonstrates the utility of low-dose prednisone in the treatment of periorbital edema in SLE. Furthermore, it illustrates how prolonged sun exposure can exacerbate SLE. Her flare was so severe that it required hospitalization with IV steroids and antibiotics. As many patients with SLE demonstrate abnormal photosensitivity, education regarding the importance of sun-protective measures is paramount.
